# Personalized transcranial alternating current stimulation improves sleep quality: Initial findings

**DOI:** 10.3389/fnhum.2022.1066453

**Published:** 2023-01-10

**Authors:** V. Ayanampudi, V. Kumar, A. Krishnan, M. P. Walker, R. B. Ivry, R. T. Knight, R. Gurumoorthy

**Affiliations:** ^1^StimScience Inc., Berkeley, CA, United States; ^2^Department of Psychology, Helen Wills Neuroscience Institute, University of California, Berkeley, Berkeley, CA, United States

**Keywords:** tACS (transcranial alternating current stimulation), sleep quality, personalization, stimulation, EEG

## Abstract

Insufficient sleep is a major health issue. Inadequate sleep is associated with an array of poor health outcomes, including cardiovascular disease, diabetes, obesity, certain forms of cancer, Alzheimer’s disease, depression, anxiety, and suicidality. Given concerns with typical sedative hypnotic drugs for treating sleep difficulties, there is a compelling need for alternative interventions. Here, we report results of a non-invasive electrical brain stimulation approach to optimizing sleep involving transcranial alternating current stimulation (tACS). A total of 25 participants (mean age: 46.3, S.D. ± 12.4, 15 females) were recruited for a null-stimulation controlled (Control condition), within subjects, randomized crossed design, that included two variants of an active condition involving 15 min pre-sleep tACS stimulation. To evaluate the impact on sleep quality, the two active tACS stimulation conditions were designed to modulate sleep-dependent neural activity in the theta/alpha frequency bands, with both stimulation types applied to all subjects in separate sessions. The first tACS condition used a fixed stimulation pattern across all participants, a pattern composed of stimulation at 5 and 10 Hz. The second tACS condition used a personalized stimulation approach with the stimulation frequencies determined by each individual’s peak EEG frequencies in the 4–6 Hz and 9–11 Hz bands. Personalized tACS stimulation increased sleep quantity (duration) by 22 min compared to a Control condition (*p* = 0.04), and 19 min compared to Fixed tACS stimulation (*p* = 0.03). Fixed stimulation did not significantly increase sleep duration compared to Control (mean: 3 min; *p* = 0.75). For sleep onset, the Personalized tACS stimulation resulted in reducing the onset by 28% compared to the Fixed tACS stimulation (6 min faster, *p* = 0.02). For a Poor Sleep sub-group (*n* = 13) categorized with Clinical Insomnia and a high insomnia severity, Personalized tACS stimulation improved sleep duration by 33 min compared to Fixed stimulation (*p* = 0.02), and 30 min compared to Control condition (*p* < 0.1). Together, these results suggest that Personalized stimulation improves sleep quantity and time taken to fall asleep relative to Control and Fixed stimulation providing motivation for larger-scale trials for Personalized tACS as a sleep therapeutic, including for those with insomnia.

## Introduction

Sleep is essential for human physical and mental health (Walker, 2021). This encompasses the homeostatic regulation of very major physiological systems within the body (e.g., cardiovascular, immune, endocrine, metabolic), as well as cognitive and emotional operations of the brain. Nevertheless, one in three adults average less than the CDC’s recommendation of at least 7 h (Centers for Disease Control and Prevention, 2022). Moreover, the life-time incidence of insomnia in general population is estimated to be over 30% (Roth, 2007; Centers for Disease Control and Prevention, 2022).

Conventional sleeping pills are widely used as therapeutic interventions for sleep difficulties. However, such sedative hypnotics have significant side effects, lack long-term efficacy, and may fail to restore normative sleep (Qaseem et al., 2016). Indeed, based on concerns of safety, efficacy, and side effects, sleeping pills are no longer recommended as a first line treatment approach for those with sleeping difficulties (Qaseem et al., 2016).

These limitations have motivated investigations of non-pharmacological approaches to enhance sleep hygiene. One approach involves non-invasive brain stimulation whereby different forms of exogenous stimulation are used to modify sleep. To date, examples of such non-invasive methods include acoustic, magnetic, and electrical stimulation approaches targeting sleep onset, sleep quality and/or sleep duration (Malkani and Zee, 2020).

The primary electrical stimulation methods are transcranial direct current stimulation (tDCS), and transcranial alternating current stimulation (tACS). Both involve the application of a low-intensity electrical current (1–2 mA) to the scalp. The main difference is that tDCS involves a constant current while tACS uses an alternating current. tACS affords the advantage of creating flexible stimulation waveforms that can be manipulated to induce modulation of neural activity in targeted frequency bands.

Electrical brain stimulation has an appealing safety profile, with no harmful adverse side effects (Bikson et al., 2004). Indeed, data collected from 33,000 sessions and over 1,000 subjects who received repeated tDCS sessions—with reports of occasional non-harmful effects such as tingling, itching, warming sensation, mild headaches, and visual flashes—indicates that tDCS is safe to use in human subjects for repeated use. Furthermore, tDCS currents of up to 2 mA have been shown to be safely tolerated (Chhatbar et al., 2017; Nitsche and Bikson, 2017). tACS has similarly been reported to be safe without any harmful adverse side effects and occasional non-harmful effects as with tDCS (Tadini et al., 2011) and has the added advantage of inducing less stimulation sensation on the scalp for the user, relative to tDCS (Fertonani et al., 2015).

Brain oscillatory patterns are grouped into different region-specific frequency bands critical for different brain functions (Basar, 1998; Canolty et al., 2006; Srinivasan et al., 2006; Thut and Miniussi, 2009; Canolty and Knight, 2010; Cohen, 2017; Haller et al., 2018). Regarding sleep, scalp EEG and intracranial EEG investigations have provided evidence that electrical signatures from the frontal lobes are useful biomarkers of sleep onset and sleep maintenance (Marzano et al., 2013; Helfrich et al., 2019). Mechanistically, it has been hypothesized that frontal lobe neurons provide “top-down” control of cortico-thalamic feedback loops involved in sleep-wake regulation (Frase et al., 2016).

Motivated by these observations, stimulation applied over the prefrontal cortex has been shown to beneficially modulate sleep (Frase et al., 2016). For example, application of 5 Hz stimulation for 10 min to the frontal cortex of subjects increased subjective sleepiness and enhances slow-frequency EEG activity in the 0.5–4.75 Hz range (D’Atri et al., 2016). Furthermore, bilateral 5 Hz tACS stimulation over fronto-temporal areas decreases sleep onset (Xie et al., 2021) and increases slow wave activity in the first half of NREM cycle relative to sham (D’Atri et al., 2019). Further studies have shown the association of alpha band activity to drowsiness and transition from wakefulness to sleepiness (Cantero et al., 2002).

To date, studies of the effect of tACS on sleep have used the same stimulation protocol for all participants, with the focusing on frequencies in the 0.5–16 Hz range (Dondé et al., 2021). However, EEG peak frequencies within specific bands show significant inter-individual differences (Salinsky et al., 1991; Zhang et al., 2021). This has motivated the recent exploration of personalized stimulation protocols that are tailored to each participant’s innate frequency profile (Okazaki et al., 2021). Indeed customized alpha or theta frequency stimulation during wakefulness, close to an individual’s internal alpha or theta frequency improves the efficacy of stimulation entrainment, measures of underlying neural plasticity, and function (Zaehle et al., 2010; Vossen et al., 2015; Vosskuhl et al., 2018; Grover et al., 2021; Huang et al., 2021; van Bueren et al., 2021).

Based on these observations, the present study sought to test the hypothesis that tACS stimulation patterns prior to sleep was effective in improving sleep, and furthermore, tested whether personalized frequency stimulation was superior to fixed frequency stimulation.

Accordingly, the study employed two tACS approaches, one using a fixed stimulation protocol and the other using an individually tailored stimulation protocol. Participants were tested in their home using a custom designed headband that provided bilateral stimulation through two frontal electrodes. Using an app-based control system, stimulation was self-administered for 15 min just before the participant went to sleep. Two specific predictions were tested: (1) both types of tACS stimulation would improve total sleep duration and the time taken to fall asleep relative to a null-stimulation control condition, and (2) personalized tACS stimulation would provide superior sleep improvement on both metrics relative to fixed tACS stimulation.

## Materials and methods

### Overview

Participants (detailed described, below) were tested over multiple sessions in their homes, self-administering the stimulation (Fixed tACS, Personalized tACS, or control) using a custom-built stimulator composed of a headband that contained two electrodes positioned over the frontal lobes. The participants used a customized phone app that randomly determined the stimulation mode for each of the alternating weeks. Sleep onset and duration were measured with a wearable tracker (Fitbit watch). The participants put the headband and tracker on when they were ready to go to sleep and used the phone app to start the stimulation.

The study protocol and an investigational device exemption for this custom stimulator were approved for this study by a 3rd party IRB.

## Participants and protocols

**FIGURE 1:**
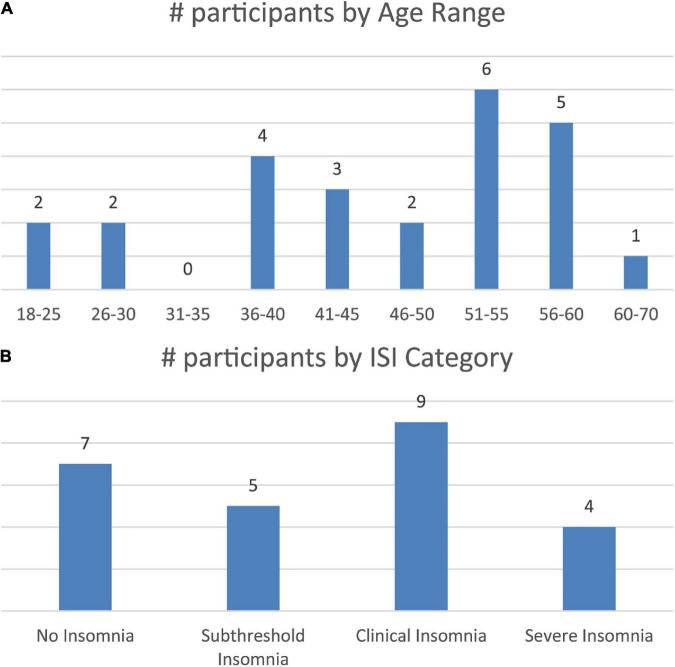
**(A)** Age distribution of the participants. **(B)** ISI distribution of participants.

A total of 25 participants were tested in a repeated-measures, cross-over design (mean age: 46.3, with the age ranging between 19 and 60, 10 male and 15 female; Figure 1A). Participants completed the Insomnia Severity Index questionnaire prior to their first session using an online form Figure 1B. There was substantive representation of the ISI categories across the participants, with 28% categorized with no insomnia, 20% with subthreshold insomnia, 36% with clinical insomnia, and 16% with severe insomnia.

**FIGURE 2:**
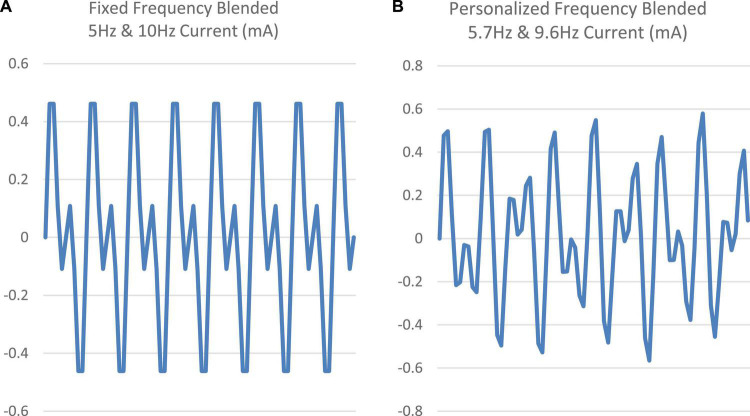
**(A,B)** Comparison of the fixed frequency (5 and 10 Hz) signal vs. a sample personalized frequency (5.7 and 9.6 Hz) signal.

For the Fixed tACS condition, a stimulation waveform composed of two sinusoids was created, one at 10 Hz (targeting the alpha band) and one at 5 Hz (targeting the theta band). The two components were started in phase and summed to a maximal possible amplitude with every other cycle of the 10 Hz signal (Figure 2A). These two frequency bands were targeted given their association with sleep onset, with alpha band activity (8–13 Hz) linked to stage I sleep and theta band activity (4–8 Hz) linked to the transition to stage II sleep (Basar, 1998). The amplitude of both sinusoids was set at 0.6 mA intensity (peak-to-peak) (Figure 2B).

For the Personalized tACS condition, a preliminary session was conducted to collect EEG data from the participants to identify, on an individual basis, the power peaks within the alpha and theta bands. These data were obtained during a 15-min daytime session, with the participant in a relaxed, eyes-closed state, before any of the pre-sleep stimulation sessions. The custom stimulator device had 2 channels of EEG with electrodes at frontal-lobe sites of Fp1 and Fp2 using a bipotential reference electrode at Fpz.

The EEG signal was band pass filtered with cutoff frequencies at 0.3 and 45 Hz. The power spectral density (PSD) was calculated using the Welch method on the filtered data and the Fooof algorithm (Haller et al., 2018) was used to determine frequency peaks after removing the aperiodic component of the spectrum. A k-means algorithm was used to calculate all peaks between 3 and 12 Hz.

Two peaks were identified from these peaks, the first selected as the peak closest to 5 Hz within 4–6 Hz band and second selected as the peak closest to 10 Hz within 9–11 Hz band. The stimulation waveform for the personalized condition was created by combining the sinusoids at the identified two peak frequencies. As with the Fixed stimulation protocol, the amplitude of both sinusoids was set to 0.6 mA (peak-to-peak). The two sinusoids in the Personalized stimulation protocol were started in phase, but they were not harmonics. Figure 2 shows the two blended signals (fixed frequency and a sample personalized frequency).

Each participant used the device over a micro-longitudinal 2-week intervention period. For one of the weeks, the app was randomly set to deliver Fixed stimulation for 15 min. For the other week, the app was set to deliver Personalized stimulation for 15 min. Participants were asked to use the headset “as often as convenient,” with the recognition that they were unlikely to use the device on each night. Days in which the participants did not wear the headset (no stimulation) or when they failed to use the app to administer stimulation served as a Control condition (with data available only if they were wearing the tracker device). On average, Fixed stimulation was administered on 5 days (range: 3–6), Personalized on 4 days (range: 3–6), and 3 days of Control data was obtained (range: 1–6).

### Data analysis

Sleep tracking data was obtained using a Fitbit tracker, the output of which provided sleep/wake durations for the participant through the night. Sleep stage data from the device were not analyzed since the classifier accuracy of different sleep stages for the Fitbit tracker are not sufficiently robust. On days with Fixed or Personalized tACS, sleep onset was defined as the interval between the end of stimulation, with the customized phone app notifying the end of stimulation, and the start of the first sleep epoch determined by the tracker data.

As stimulation sensation was noticeable for the participant, we used the end of stimulation as the event from which to begin measuring sleep onset. Given that there was no stimulation in the Control condition, sleep onset data (as defined to be measured from the end of stimulation) was not recordable for this condition.

Data outliers were defined based on two pre-hoc criteria: (1) If the sleep start time for a session was beyond ± 1.5 h of their sleep start time distribution inter-quartile-range, or (2) the participant’s sleep duration fell beyond ± 1.5 h of their sleep duration distribution inter-quartile-range. On average, 0.3 sessions (range: 0–2) were excluded and the distribution was similar across the conditions. From the remaining data, mean sleep onset and sleep duration scores for each participant were calculated in each condition. Note that the Control data were collected across the 2-week study period.

## Results

### Personalized vs. fixed tACS stimulation

**FIGURE 3:**
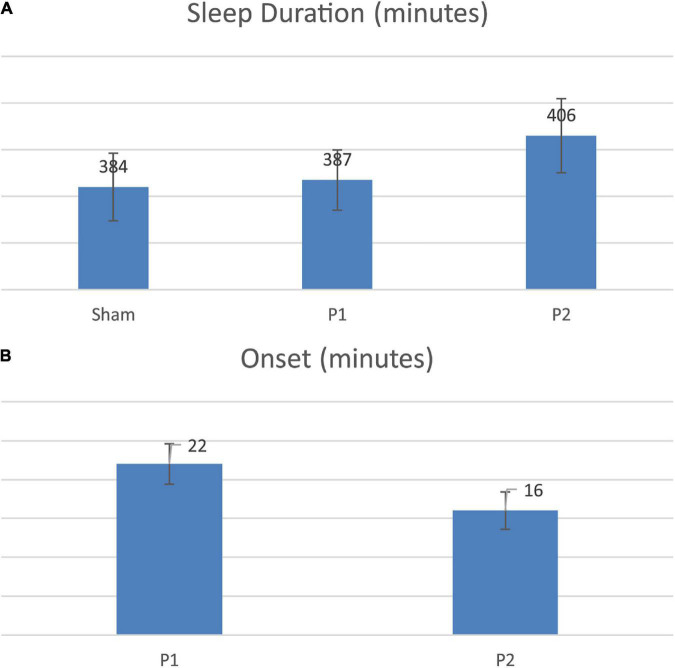
**(A)** Sleep duration comparison across conditions. **(B)** Onset comparison across stimulations.

Personalized tACS stimulation increased sleep duration by 22 min compared to the Control condition (*p* = 0.04) and 19 min compared to Fixed stimulation (*p* = 0.03; see Figure 3A). Fixed stimulation increased sleep duration by 3 min compared to Control condition, but this difference was not significant (mean: 3 min; *p* = 0.75). Personalized stimulation resulted in a faster sleep onset by 6 min compared to the Fixed stimulation (28% improvement, *p* = 0.02, refer to Figure 3B and Tables 1, 2).

**TABLE 1:** Sleep duration and onset performance.

	Personalized (P2)–Fixed (P1)	
	**Sleep duration**	**Onset**
Mean	19	–6
*P*-value	0.03	0.02

**TABLE 2:** Sleep duration compared to sham.

	Sleep duration compared to sham	
	**Fixed (P1)**	**Personalized (P2)**
Mean	3	22
*P*-value	0.75	0.04

Using the demographic information collected concerning age and sleep hygiene, two secondary analyses were conducted on sleep duration (Table 3). For the age analysis, we divided the participants into two groups based on age: ≤ 50 years old with *n* = 13, > 50 years old with *n* = 12.

**TABLE 3:** Sleep duration by age and ISI.

	Personalized (P2)–Fixed (P1)			
	**Young (<50 years)**	**Old (>50 years)**	**ISI 1 or 2**	**ISI 3 or 4**
Mean	27	10	4	33
*P*-value	0.02	0.45	0.67	0.02

### Age analysis

**FIGURE 4:**
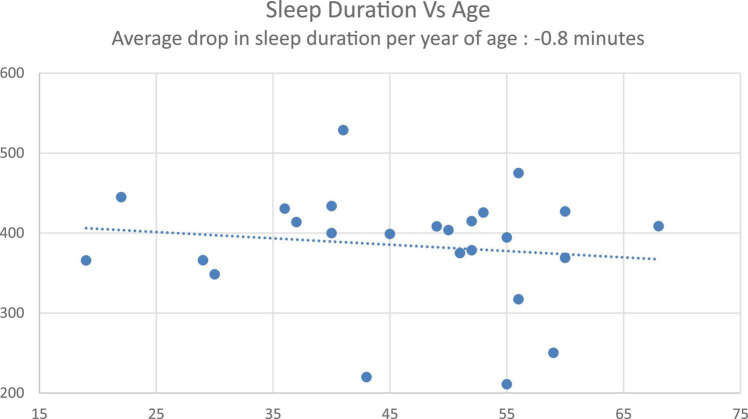
Sleep duration vs. age regression.

First, to confirm the classic age-related decrease in sleep as a validation of the cohort and its normative sleep, there was a significant correlation between age and sleep duration (data from Control condition, *r* = –0.19, *p* < 0.001), with an average –0.8-min decrease in sleep duration with every year increase in age (Figure 4), consistent with published norms (Hublin et al., 2020).

Segmenting the cohort based on age, and first focusing on the younger cohort (≤ 50 years old), Personalized tACS stimulation resulted in a 27-min increase in sleep duration, relative to Fixed tACS stimulation (*p* = 0.02), and a 29-min increase in sleep duration compared to the Control condition (*p* = 0.02). In the older cohort (> 50 years old), Personalized tACS stimulation elicited a non-significant 10-min increase in sleep duration, relative to Fixed tACS stimulation (*p* = 0.4: ns) and an also non-significant 14-min increase relative to Control condition (*p* = 0.45: ns).

Poor- vs. Good-sleeper Analyses: Based on sleep hygiene, the cohort was further segmented *post hoc* into two groups: a normative sleep group (those with no-insomnia and subclinical threshold insomnia ISI categorization, *n* = 12), and a poor sleep group (those with clinical insomnia and severe insomnia ISI categorization, *n* = 13). For the poor sleep group, Personalized tACS stimulation improved sleep duration by 33 min compared to Fixed tACS stimulation (*p* = 0.02), and 30 min sleep duration compared to Control condition (*p* < 0.1: ns). For the normative sleep group, Personalized tACS stimulation increased sleep duration by 4 min relative to Fixed tACS stimulation (*p* = 0.67; ns) and increased 13 min compared to Control condition (*p* = 0.2; ns).

## Discussion

To date, a number of studies have provided evidence that external, non-invasive stimulation of varied forms can enhance sleep quality (Marzano et al., 2013; D’Atri et al., 2016, 2019; Frase et al., 2016). For modulating electrical brain activity, these include tDCS, Tacs, and rTMS, all applied in an open loop i.e., fixed-stimulation manner, and all applied during sleep. Indeed, tACS and rTMS protocols targeting oscillatory patterns in different frequency bands (0.5–16 Hz) using fixed stimulation waveforms across all subjects have led to improvements in several sleep metrics (Ngo et al., 2013; Oroz et al., 2021).

The current study took a different approach. Specifically, the study tested whether tACS before (rather than during) sleep would similarly improve sleep, and furthermore whether a novel personalized tACS stimulation would further augment sleep effects compared to fixed tACS stimulation.

Comparing the two active stimulation conditions, the results suggest that the Personalized tACS stimulation improves sleep duration and sleep onset relative to Fixed tACS stimulation (19 more minutes of sleep and 6 min earlier onset of sleep).

An age-related analysis validated the known sleep norms of sleep duration decreasing with age (decrease of 0.8 min per year observed with the Control condition). Previous studies have shown that night-to-night and inter-participant variability in sleep quality increases in older cohorts (Buysse et al., 2010). Given these norms of aging and sleep quality deterioration, we performed a *post hoc* analysis of the impact of Personalized tACS stimulation on sleep duration relative to Control condition, for a younger cohort (≤50 years old) and an older cohort (> 50 years old). We observed a robust 29-min increase in sleep duration for the younger cohort. For the older cohort, there was a non-significant sleep duration increase of 14-min, providing a potential for using the Personalized tACS stimulation to improve sleep quality with age. Given this increase was not significant, it requires further exploration with a larger aging cohort (current effect size is 0.45 for the younger cohort, and 0.13 for the older cohort).

In addition to the age factor, insomnia symptomatology was further analyzed. This was motivated by the factor that current sedative hypnotics have non-trivial side effects, have challenging aspects of long-term efficacy, may fail to implement normative sleep physiology (Qaseem et al., 2016), and the recent American College of Physicians recommendation that they should no longer be a first line treatment approach for those with sleeping difficulties (Qaseem et al., 2016).

*Post hoc* analysis demonstrated that the poor sleep sub-group (defined as having significant insomnia categorization using the ISI) showed a larger boost in sleep duration (33 min increase with an effect size of 0.55) from personalized tACS stimulation compared to the Fixed tACS protocol. As a point of contrast, the typical prescription sleep medication, zolpidem (brand name, Ambien), has been shown to increase total sleep time by 35.5 min relative to placebo (Randall et al., 2012), and a more recent medication, suvorexant (brand name, Belsomra), has a reported increase in total sleep time of 28 min (Clinical Trials, 2019). As such, the current results offer tentative evidence that non-invasive, personalized stimulation may be a viable alternative intervention for insomnia, should such finding be replicated large-scale.

Finally, the current study administered the tACS stimulation for 15-min pre-sleep and involved no additional stimulation during sleep. With participants aware of stimulation sensations, this approach has been selected based on multiple studies showing that the benefits of stimulation last well beyond the stimulation period (Thut et al., 2011; Kasten et al., 2016; He et al., 2020). That said, we can see two important extensions of the current approach in future studies. First, resting state EEG could be obtained prior to each night to account for within-subject fluctuations across days in peak frequency. Second, EEG could be monitored during sleep to allow for additional personalized stimulation during the night.

### Limitations

The sample size of 25 in the present study is modest, especially when considering the large age range, variance in ISI scores, and the fact that participants self-administered tACS stimulation in their home setting at a time of night that was variable. Clearly this work needs to be replicated and extended in studies with samples of significantly greater size, and within targeted subgroups (e.g., age, sleep hygiene).

In the Fixed tACS stimulation, the two frequencies (5 and 10 Hz) are harmonics. Thus, this condition differs from the Personalized in the phase or temporal coherence of the composite waveform given that there will be a synchronized maximum every 2 cycles of the 10 Hz. This pattern is not present in the Personalized tACS stimulation. Given that the Fixed condition failed to produce a benefit, it may be that having a consistent phase relationship may have offset the effects of the base frequencies. This issue can be addressed in future work by using a fixed pattern in which the two components are not harmonics. It would also be useful to compare two individualized stimulation patterns assigned to each individual, one based on their own EEG recordings and a control condition involving another individual’s EEG recordings.

The null-stimulation Control condition in this current study did not represent a classical sham stimulation (which typically involves a short ramp-up period of stimulation lasting approximately 15 s and ramp-down). While this is an experimental weakness, it is important to recognize that participants are unlikely to be fully blinded with our current mode of stimulation; in general, participants were aware of sensation when the stimulation was on. In addition, the number of nights with null-stimulation were lower than the stimulation condition nights leading to a lower signal-to-noise ratio. Nonetheless, a typical balanced sham control should be implemented in future studies.

Given previous results using tACS in the alpha and theta range, we were surprised that the Fixed tACS stimulation failed to produce a significant improvement on the sleep measures compared to the Control condition (effect size of 0.3). This null result could have resulted from a lack of power in terms of sample size within the sub-cohorts including the control condition or some of the other factors discussed above (e.g., use of a composite involving harmonics). Nonetheless, the potential of non-invasive brain stimulation is supported by our finding that personalized tACS stimulation provided significant improvements relative to fixed tACS stimulation and the control condition.

## Conclusion

These findings provide evidence that frequency specific personalized electrical brain stimulation offers a promising method for optimizing human sleep quality. Moreover, these effects were especially prominent in those with the highest ratings of insomnia, suggesting a potential future therapeutic opportunity, once there is replication of these findings in a larger cohort.

## Data availability statement

The original contributions presented in this study are included in the article/supplementary material, further inquiries can be directed to the corresponding author.

## Ethics statement

The studies involving human participants were reviewed and approved by E&I Review. The patients/participants provided their written informed consent to participate in this study.

## Author contributions

RG contributed the conception or design of the work, acquisition, analysis and interpretation of data, and manuscript writing, revision, and approval. VA contributed to acquisition, analysis, and interpretation of data, and writing the first draft of the manuscript. VK contributed to acquisition, analysis, and interpretation of data. AK contributed to the interpretation of data and writing the first draft of the manuscript. RK, MW, and RI contributed the conception or design of the work, analysis and interpretation of data, and manuscript writing, revision, and approval. All authors contributed to the article and approved the submitted version.

## References

[B1] Basar E. (1998). *Brain function and oscillations: Principles and approaches.* Berlin: Springer.

[B2] Bikson M. Inoue M. Akiyama H. Deans J. K. Fox J. E. Miyakawa H. (2004). Effects of uniform extracellular DC electric fields on excitability in rat hippocampal slices *in vitro*. *J. Physiol.* 557(Pt 1), 175–190. 10.1113/jphysiol.2003.055772 14978199 PMC1665051

[B4] Buysse D. Cheng Y. Germain A. Moul D. E. Franzen P. L. Fletcher M. (2010). Night-to-night sleep variability in older adults with and without chronic insomnia. *Sleep Med.* 11 56–64. 10.1016/j.sleep.2009.02.010 19962939 PMC2818595

[B5] Canolty R. T. Knight R. T. (2010). The functional role of cross-frequency coupling. *Trends Cogn. Sci*. 14 506–515. 10.1016/j.tics.2010.09.001 20932795 PMC3359652

[B6] Canolty R. T. Edwards E. Dalal S. S. Soltani M. Nagarajan S. S. Kirsch H. E. (2006). High gamma power is phase-locked to theta oscillations in human neocortex. *Science* 313 1626–1628. 10.1126/science.1128115 16973878 PMC2628289

[B7] Cantero J. Atienza M. Salas R. (2002). Human alpha oscillations in wakefulness, drowsiness period, and REM sleep: Different electroencephalographic phenomena within the alpha band. *Clin. Neurophysiol.* 32 54–71. 10.1016/s0987-7053(01)00289-1 11915486

[B8] Centers for Disease Control and Prevention (2022). *Sleep and sleep disorders: Data and statistics.* Available online at: https://www.cdc.gov/sleep/data_statistics.html (accessed June 6, 2022).

[B9] Chhatbar P. Y. Chen R. Deardorff R. Dellenbach B. Kautz S. A. George M. S. (2017). Safety and tolerability of transcranial direct current stimulation to stroke patients - A phase I current escalation study. *Brain Stimul.* 10 553–559. 10.1016/j.brs.2017.02.007 28279641 PMC5411981

[B10] Clinical Trials (2019). *Merck’s Belsomra achieves Phase III efficacy endpoints for insomnia.* Available online at: https://www.clinicaltrialsarena.com/news/merck-belsomra-phase-iii-results/ (accessed August 15, 2022).

[B11] Cohen M. (2017). Where does EEG come from and what does it mean? *Trends Neurosci.* 40 208–218. 10.1016/j.tins.2017.02.004 28314445

[B12] D’Atri A. De Simoni E. Gorgoni M. Ferrara M. Ferlazzo F. Rossini P. M. (2016). Electrical stimulation of the frontal cortex enhances slow-frequency Eeg activity and sleepiness. *Neuroscience* 324 119–130. 26964682 10.1016/j.neuroscience.2016.03.007

[B13] D’Atri A. Scarpelli S. Gorgoni M. Alfonsi V. Annarumma L. Giannini A. M. (2019). Bilateral Theta Transcranial Alternating Current Stimulation (tACS) Modulates EEG Activity: When tACS works awake it also works asleep. *Nat. Sci. Sleep* 11 343–356. 10.2147/NSS.S229925 31819688 PMC6875492

[B14] Dondé C. Brunelin J. Micoulaud-Franchi J. Maruani J. Lejoyeux M. Polosan M. (2021). The effects of transcranial electrical stimulation of the brain on sleep: A systematic review. *Front. Psychiatry* 12:646569. 10.3389/fpsyt.2021.646569 34163380 PMC8215269

[B15] Fertonani A. Ferrari C. Miniussi C. (2015). What do you feel if I apply transcranial electric stimulation? Safety, sensations and secondary induced effects. *Clin. Neurophysiol.* 126 2181–2188. 10.1016/j.clinph.2015.03.015 25922128

[B16] Frase L. Piosczyk H. Zittel S. Jahn F. Selhausen P. Krone L. (2016). Modulation of total sleep time by Transcranial Direct Current Stimulation (tDCS). *Neuropsychopharmacology* 41 2577–2586. 10.1038/npp.2016.65 27143601 PMC4987856

[B17] Grover S. Nguyen J. Reinhart R. (2021). Synchronizing brain rhythms to improve cognition. *Annu. Rev. Med.* 72 29–43. 10.1146/annurev-med-060619-022857 33035432 PMC10068593

[B18] Haller M. Donoghue T. Peterson E. Varma P. Sebastian P. Gao R. (2018). Parametrizing neural power spectra. *bioRxiv* [Preprint]. 10.1101/299859 PMC8106550 33230329

[B19] He W. Fong P. Leung T. Huang Y. (2020). Protocols of non-invasive brain stimulation for neuroplasticity induction. *Neurosci. Lett.* 719:133437. 10.1016/j.neulet.2018.02.045 29476796

[B20] Helfrich R. F. Lendner J. D. Mander B. A. Guillen H. Paff M. Mnatsakanyan L. (2019). Bidirectional prefrontal-hippocampal dynamics organize information transfer during sleep in humans. *Nat. Commun.* 10:3572. 10.1038/s41467-019-11444-x 31395890 PMC6687745

[B21] Huang W. Stitt I. Negahbani E. Passey D. Ahn S. Davey M. (2021). Transcranial alternating current stimulation entrains alpha oscillations by preferential phase synchronization of fast-spiking cortical neurons to stimulation waveform. *Nat. Commun.* 12:3151. 10.1038/s41467-021-23021-2 34035240 PMC8149416

[B22] Hublin C. Haasio L. Kaprio J. (2020). Changes in self-reported sleep duration with age - a 36-year longitudinal study of Finnish adults. *BMC Public Health* 20:1373. 10.1186/s12889-020-09376-z 32907578 PMC7487757

[B23] Kasten F. H. Dowsett J. Herrmann C. S. (2016). Sustained aftereffect of α-tACS Lasts Up to 70 min after Stimulation. *Front. Hum. Neurosci.* 10:245. 10.3389/fnhum.2016.00245 27252642 PMC4879138

[B25] Malkani R. Zee P. (2020). Brain stimulation for improving sleep and memory. *Sleep Med. Clin.* 15 101–115. 10.1016/j.jsmc.2019.11.002 32005347

[B26] Marzano C. Moroni F. Gorgoni M. Nobili L. Ferrara M. De Gennaro L. (2013). How we fall asleep: Regional and temporal differences in electroencephalographic synchronization at sleep onset. *Sleep Med.* 14 1112–1122. 10.1016/j.sleep.2013.05.021 24051119

[B27] Ngo H.-V. V. Martinetz T. Born J. Mölle M. (2013). Auditory closed-loop stimulation of the sleep slow oscillation enhances memory. *Neuron* 78 545–553. 10.1016/j.neuron.2013.03.006 23583623

[B28] Nitsche M. Bikson M. (2017). Extending the parameter range for tDCS: Safety and tolerability of 4 mA stimulation. *Brain Stimul.* 10 541–542. 10.1016/j.brs.2017.03.002 28456325 PMC5972544

[B29] Okazaki Y. Nakagawa Y. Mizuno Y. Hanakawa T. Kitajo K. (2021). Frequency- and area-specific phase entrainment of intrinsic cortical oscillations by repetitive transcranial magnetic stimulation. *Front. Hum. Neurosci.* 15:608947. 10.3389/fnhum.2021.608947 33776666 PMC7994763

[B30] Oroz R. Kung S. Croarkin P. Cheung J. (2021). Transcranial magnetic stimulation therapeutic applications on sleep and insomnia: A review. *Sleep Sci. Pract.* 5:3. 10.1186/s41606-020-00057-9

[B31] Qaseem A. Kansagara D. Forciea M. Cooke M. Denberg T. Clinical Guidelines Committee of the American College of Physicians. (2016). Management of chronic insomnia disorder in adults: A clinical practice guideline from the American college of physicians. *Ann. Intern. Med.* 165 125–133. 10.7326/M15-2175 27136449

[B32] Randall S. Roehrs T. Roth T. (2012). Efficacy of eight months of nightly Zolpidem: A prospective placebo-controlled study. *Sleep.* 35 1551–1557. 10.5665/sleep.2208 23115404 PMC3466802

[B33] Roth T. (2007). Insomnia: Definition, prevalence, etiology, and consequences. *J. Clin. Sleep Med.* 3 (Suppl. 5), S7–S10. 17824495 PMC1978319

[B34] Salinsky M. C. Osken B. S. Morehead L. (1991). Test-retest reliability in EEG frequency analysis. *Electroencephalogr. Clin. Neurophysiol.* 79 382–392. 10.1016/0013-4694(91)90203-g 1718711

[B35] Srinivasan R. Winter W. Nunez P. (2006). Source analysis of EEG oscillations using high resolution EEG and MEG. *Prog. Brain Res.* 159 29–42. 10.1016/S0079-6123(06)59003-X 17071222 PMC1995013

[B36] Tadini L. El-Nazer R. Brunoni A. R. Williams J. Carvas M. Boggio P. (2011). Cognitive, mood, and electroencephalographic effects of noninvasive cortical stimulation with weak electrical currents. *JECT* 27 134–140. 10.1097/YCT.0b013e3181e631a8 20938352

[B37] Thut G. Miniussi C. (2009). New insights into rhythmic brain activity from TMS–EEG studies. *Trends Cogn. Sci.* 182–189. 10.1016/j.tics.2009.01.004 19286414

[B38] Thut G. Schyns P. G. Gross J. (2011). Entrainment of perceptually relevant brain oscillations by non-invasive rhythmic stimulation of the human brain. *Front. Psychol.* 2:170. 10.3389/fpsyg.2011.00170 21811485 PMC3142861

[B39] van Bueren N. Reed T. Nguyen V. Sheffield J. van der Ven S. Osborne M. (2021). Personalized brain stimulation for effective neurointervention across participants. *PLoS Comput. Biol.* 17:e1008886. 10.1371/journal.pcbi.1008886 34499639 PMC8454957

[B40] Vossen A. Gross J. Thut G. (2015). Alpha power increase after transcranial alternating current stimulation at alpha frequency reflects plastic changes rather than entrainment. *Brain Stimul.* 8 499–508. 10.1016/j.brs.2014.12.004 25648377 PMC4464304

[B41] Vosskuhl J. Strüber D. Herrmann C. S. (2018). Non-invasive brain stimulation: A paradigm shift in understanding brain oscillations. *Front. Hum. Neurosci.* 12:211. 10.3389/fnhum.2018.00211 29887799 PMC5980979

[B42] Walker M. P. (2021). Sleep essentialism. *Brain* 144 697–699. 10.1093/brain/awab026 33787879

[B43] Xie J. T. Wang L. Xiao C. Ying S. Ren J. Chen Z. (2021). Low Frequency transcranial alternating current stimulation accelerates sleep onset process. *IEEE Trans. Neural Syst. Rehabil. Eng.* 29 2540–2549. 10.1109/TNSRE.2021.3131728 34851828

[B44] Zaehle T. Rach S. Hermann C. (2010). Transcranial alternating current stimulation enhances individual alpha activity in human EEG. *PLoS One* 5:e13766. 10.1371/journal.pone.0013766 21072168 PMC2967471

[B45] Zhang Y. Lu Y. Wang D. Zhou C. Xu C. (2021). Relationship between individual alpha peak frequency and attentional performance in a multiple object tracking task among ice-hockey players. *PLoS One* 16:e0251443. 10.1371/journal.pone.0251443 34043652 PMC8158945

